# Supplementary material for: The enterovirus genome can be translated in an IRES-independent manner that requires the initiation factors eIF2A/eIF2D
Source: PLoS Biol. 2023 Jan 23;21(1):e3001693. doi: 10.1371/journal.pbio.3001693 (PMC9894558; doi:10.1371/journal.pbio.3001693)
Supplement: S1 Table — (DOCX) [file pbio.3001693.s001.docx]

**S1 Table. Oligonucleotides used in the study**

PV

| **Oligonucleotide** | **Sequence (5' - 3')** |
| --- | --- |
| 2C BmgBI F | AGCTGAAAGAGAAAACACGTCCAC |
| 3D BglII R | CTTTGGCATCCAAGATCTCCACTTC |
| 3B STOP F | GTACAAGGACCAGGGTTCGATTAgtaAGTGGCTATGGCTAAAAG |
| 3B STOP R | CTTTTAGCCATAGCCACTtacTAATCGAACCCTGGTCCTTGTAC |
| Δ SLII-3 gBlock | CGAAAACCCGGTATCCCGGGTTCTTAAAACAGCTCTGGGGTTGTACCCACCC  CAGAGGCCCACGTGGCGGCTAGTACTCCGGTATTGCGGTACCCTTGTACGCC  TGTTTTATACTCCCTTCCCGTAACTTAGACGCACAAAACCAAGTTCAATAGA  AGG-----CAAACCAG-----CCACGAACAAGCACTTCTGTTTCCCCGGTGA  TGTCGTATAGACTGCTTGCGTGGTTGAAAGCGACGGATCCGTTATCCGCTTA  TGTACTTCGAGAAGCCCAGTACCACCTCGGAATCTTCGATGCGTTGCGCTCA  GCACTCAACCCCAGAGTGTAGCTTAGGCTGATGAGTCTGGACATCCCTCACC  GGTGACGGTGGTCCAG |
| 3D BglII F | GGCAAAGAAGTGGAGATCTTGGATGC |
| 3D ApaI R | AATTAATTCATCGATGAATTCGGGCC |
| XhoI F | GTCCAAATTGCTCGAGGATC |
| SpeI R | GTTCCTAGTTATAATAACTAGTGAG |
| 2A STOP F | GAACAAGGCATCACCtAgTAaATAGAGTCACTTG |
| 2A STOP R | CAAGTGACTCTATtTAcTaGGTGATGCCTTGTTC |
| Δ AUG F | GAAGAAGCC---GAACAAGGCATCACCAAT |
| Δ AUG R | GCCTTGTTC---GGCTTCTTCTTCGTAGGC |
| UUG / CUG / CUA / CUC F | GAAGAAGAAGCCATGGAACAAGGCATC  tTG/cTG/cTa/cTc |
| UUG / CUG / CUA / CUC R | GATGCCTTGTTCCATGGCTTCTTCTTC  CAa/CAg/tAg/gAg |
| NanoLuc gBlock | GGACCACTCCAGGGCGGAGGTGGGGGAGGTGAATTCATGGTCTTCACACTGG  AAGATTTTGTTGGGGACTGGAGGCAGACAGCAGGCTACAACCTGGACCAAGT  CCTTGAACAGGGTGGTGTGTCCAGTCTTTTTCAGAATCTGGGGGTGTCTGTG  ACTCCAATCCAAAGGATTGTCCTGAGTGGTGAAAATGGGCTGAAGATTGACA  TCCATGTCATCATCCCTTATGAAGGTCTGTCTGGTGACCAAATGGGCCAGAT  TGAAAAAATTTTCAAGGTGGTGTACCCTGTGGATGATCATCACTTCAAGGTG  ATCCTGCACTATGGCACACTGGTCATTGATGGGGTCACCCCAAACATGATTG  ACTATTTTGGCAGGCCTTATGAAGGCATTGCTGTGTTTGATGGCAAAAAGAT  CACTGTGACAGGGACCCTGTGGAATGGCAACAAAATCATTGATGAGAGGCTG  ATCAACCCAGATGGCTCCCTGCTGTTCAGGGTCACCATCAATGGTGTGACAG  GCTGGAGGCTGTGTGAAAGGATTCTGGCTCTCGAGGAGGCTTTGTTTCAA |
| 2C Linker R | TAGAGGACCCTGGAATAATGCTTCCATACAATTGCCAATG |
| Linker NanoLuc F | TTATTCCAGGGTCCTCTACAAGGCGGAGGTGGGGGAGGTG |
| NanoLuc 3A R | GAGTGGTCCTTGAAACAAAGCCTCCTC |
| NanoLuc 3A F | CTTTGTTTCAAGGACCACTCCAGTATAAAG |

EV-A71

| BstEI EV-A71 F | CTGCGATATTGAGGTGACCGA |
| --- | --- |
| SpeI EV-A71 R | GCCTTGGGTCTTTACTAGTCA |
| 3B STOP EV-A71 F | GTGCAGGGGCCGAGCtAgTaATTCGCCCTATCTCTA |
| 3B STOP EV-A71 R | TAGAGATAGGGCGAATtAcTaGCTCGGCCCCTGCAC |
| SpeI EV-A71 F | CAGCAGTGCTGACTAGTAAAG |
| AfeI EV-A71 R | CAGGGTCAATGCCAGCGCTTC |
| SalI EV-A71 R | CTCTCAAGGGCATCGGTCGACAG |
| 3D GAA EV-A71 F | CATGGTGGCCTACGGGGcTGcTGTGTTGGCTAGTTAC |
| 3D GAA EV-A71 R | GTAACTAGCCAACACAgCAgCCCCGTAGGCCACCATG |
