# Supplementary material for: The enterovirus genome can be translated in an IRES-independent manner that requires the initiation factors eIF2A/eIF2D
Source: PLoS Biol. 2023 Jan 23;21(1):e3001693. doi: 10.1371/journal.pbio.3001693 (PMC9894558; doi:10.1371/journal.pbio.3001693)
Supplement: S1 Raw Images — (PDF) [file pbio.3001693.s003.pdf]

Raw uncropped images for Fig. 8 A-D

Activation of PKR and phosphorylation of eIF2α phosphorylation in response to transfection of PV subgenomic replicon RNA. Western blot analysis of p-PKR (T446) (panel **A**), PKR (panel **B**), p-eIF2α (S51) (panel **C**), and eIF2α (panel **D**) in HeLa cell lysates. Cells were transfected individually with PV subgenomic replicon RNAs: ∆IRES, 3B STOP, and WT or with WT in the presence of 3 mM GuHCl (WT_GuHCl). Six hours post-transfection, cells were processed for western blot analysis and probed using anti-p-PKR (T446), PKR, p-eIF2α (S51), and eIF2α antibodies; α/βtubulin was used as a loading control.

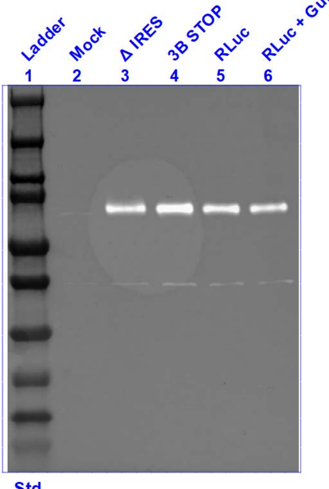
Ladder
Mock
Δ IRES
3B STOP
RLuc
RLuc + Gu
1
2
3
4
5
6
Std

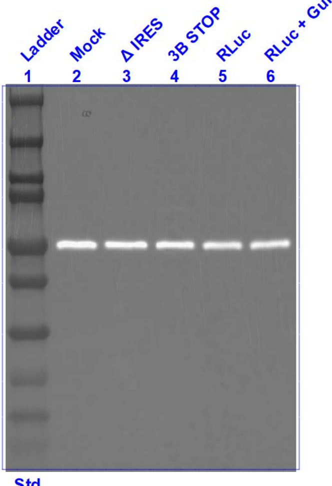
Ladder
Mock
Δ IRES
3B STOP
RLuc
RLuc + Gu
1
2
3
4
5
6
Std

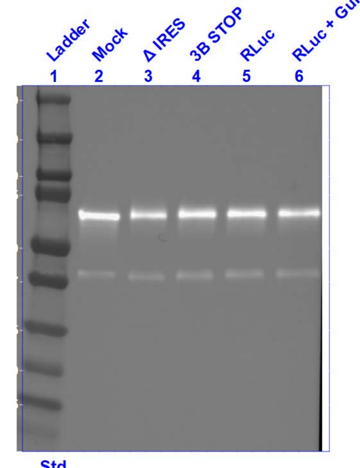

Ladder
Mock
Δ IRES
3B STOP
RLuc
RLuc + Gu
1
2
3
4
5
6
Std

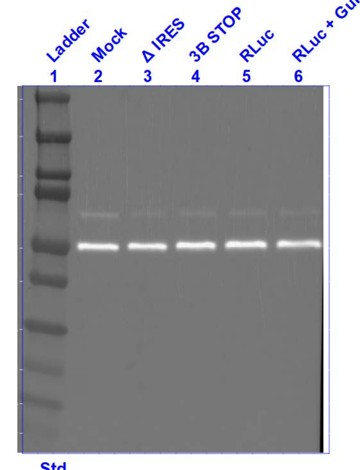
Ladder
Mock
Δ IRES
3B STOP
RLuc
1
2
3
4
5
6
Std

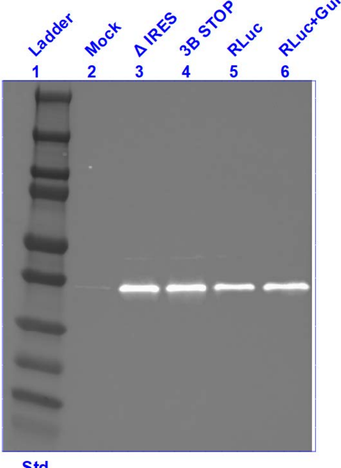
Ladder
Mock
Δ IRES
3B STOP
RLuc
RLuc+Gu
1
2
3
4
5
6
Std

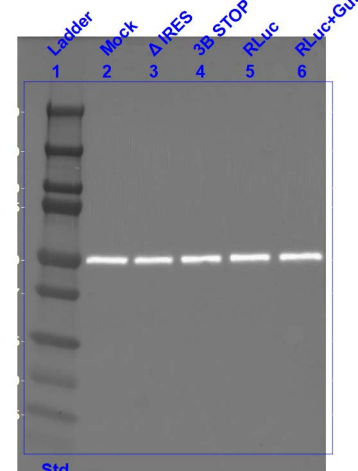

Ladder
Mock
Δ IRES
3B STOP
RLuc
1
2
3
4
5
6
Std

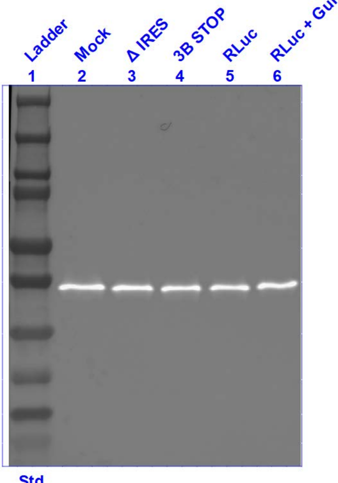
Ladder
Mock
Δ IRES
3B STOP
RLuc
RLuc + Gu
1
2
3
4
5
6
Std

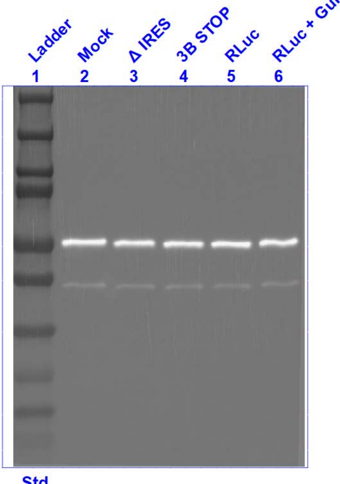
Ladder
Mock
Δ IRES
3B STOP
RLuc
RLuc + Gu
1
2
3
4
5
6
Std

Original Blots

RAW BLOT Figure 12E. RAW BLOT Figure 12F.

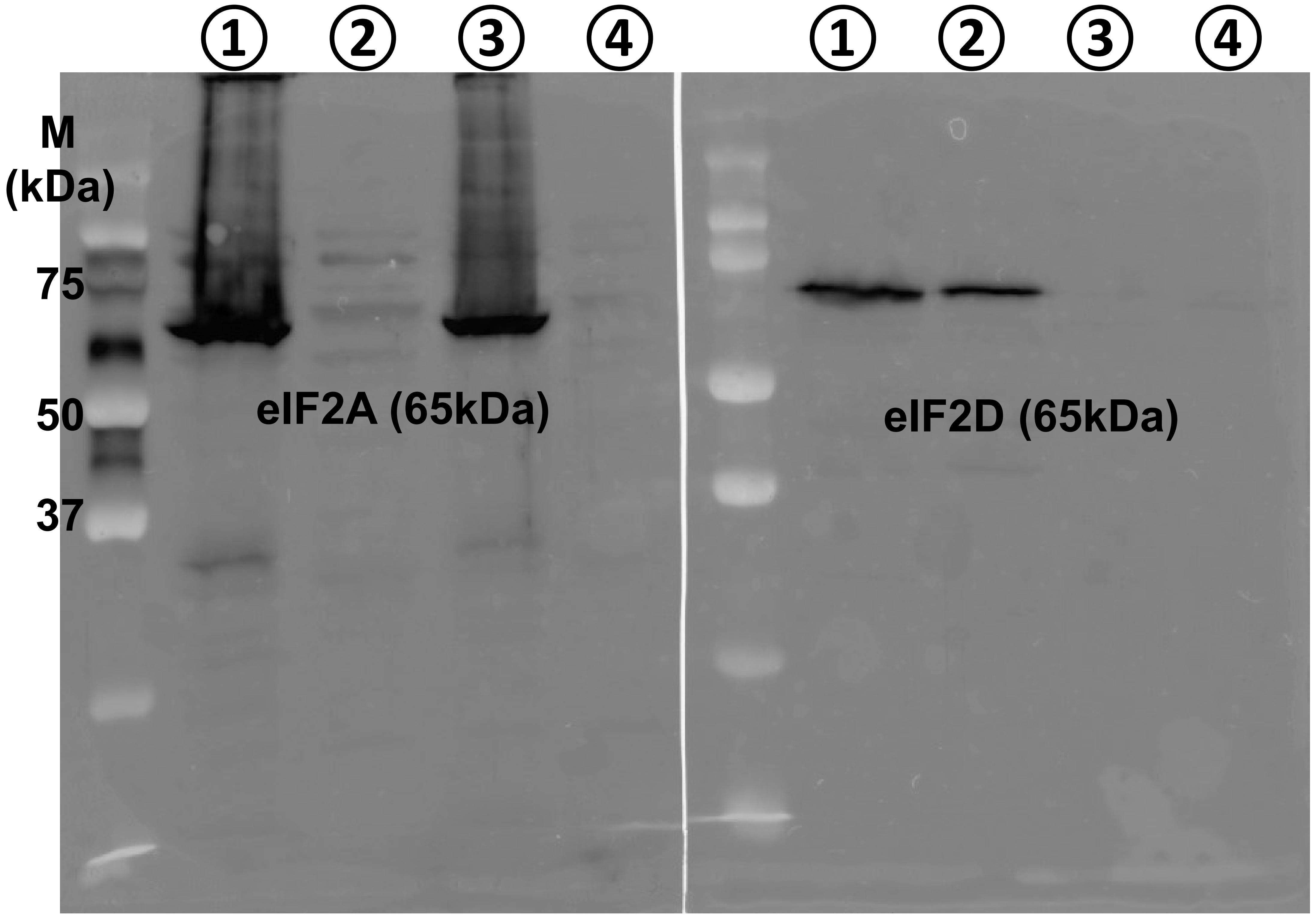

RAW BLOT Figure 12G.

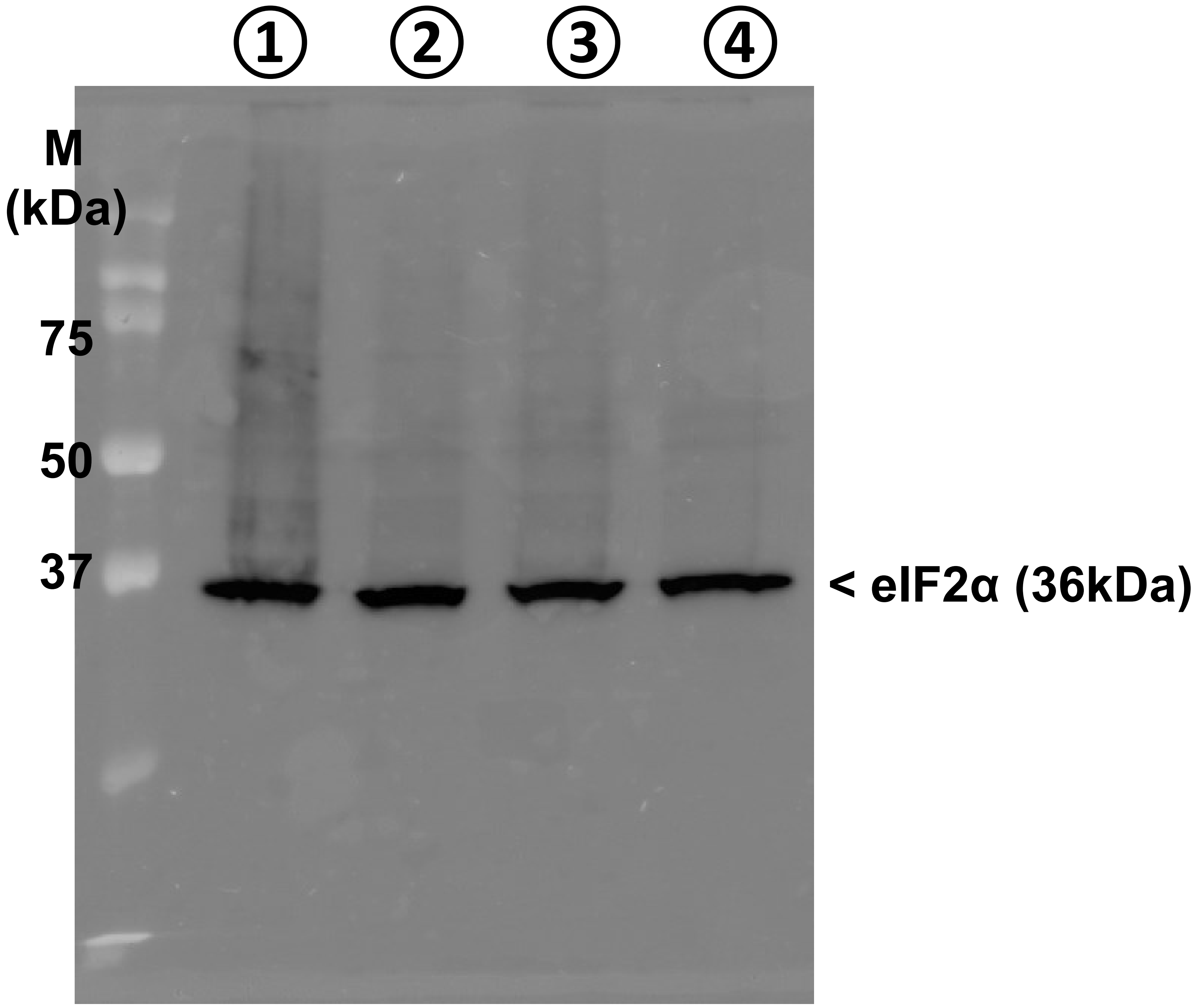

HAP1 ① WT, ② eIF2A-KO, ③ eIF2D-KO, ④ eIF2A-KO / eIF2D-KO cells

10^6 Cells loaded in each lane in 12.5% gel

First probed for anti-eIF2A, or -eIF2D, or -eIF2α antibodies.

Original Blots

RAW BLOT Figure 12E. RAW BLOT Figure 12F.

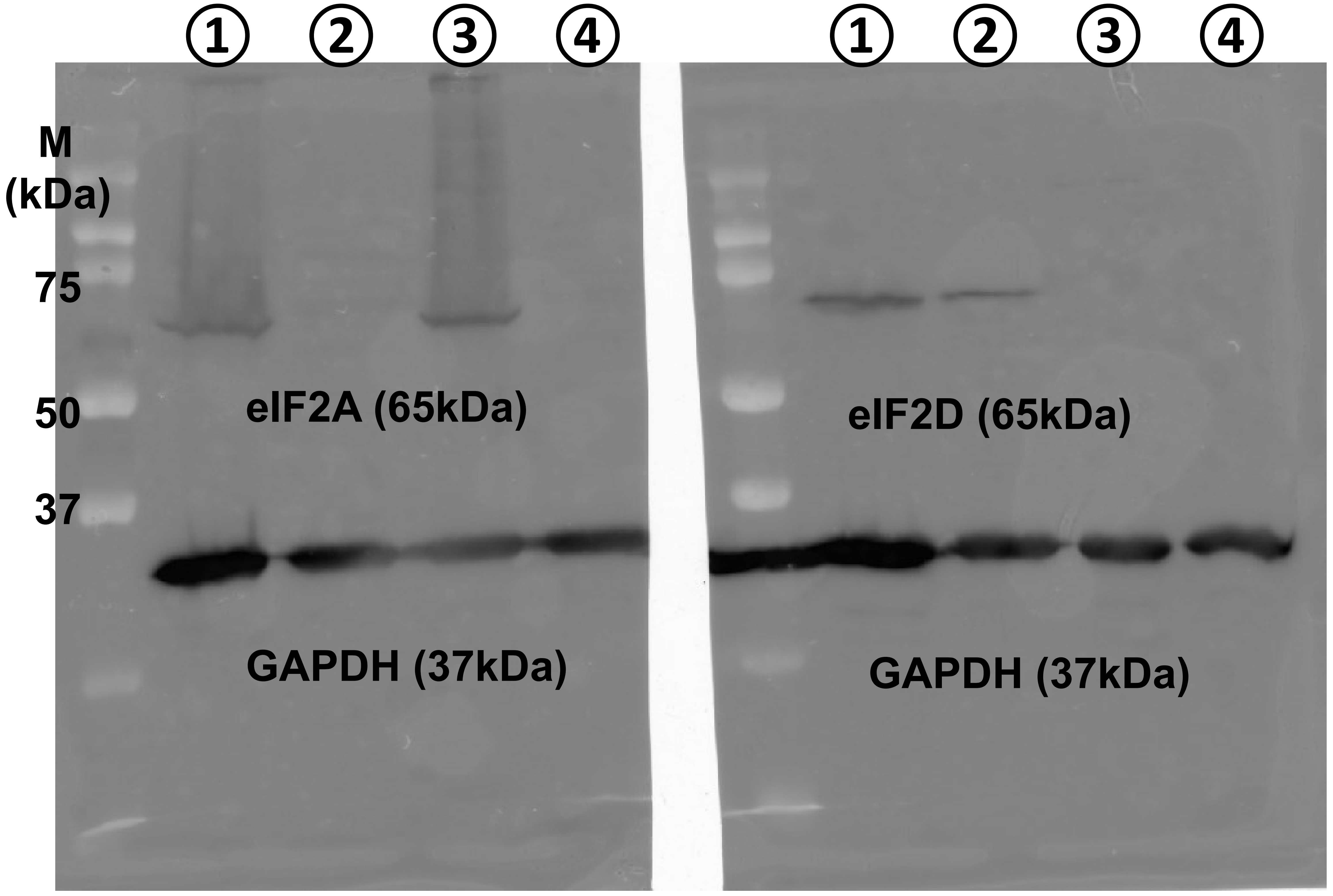

RAW BLOT Figure 12G.

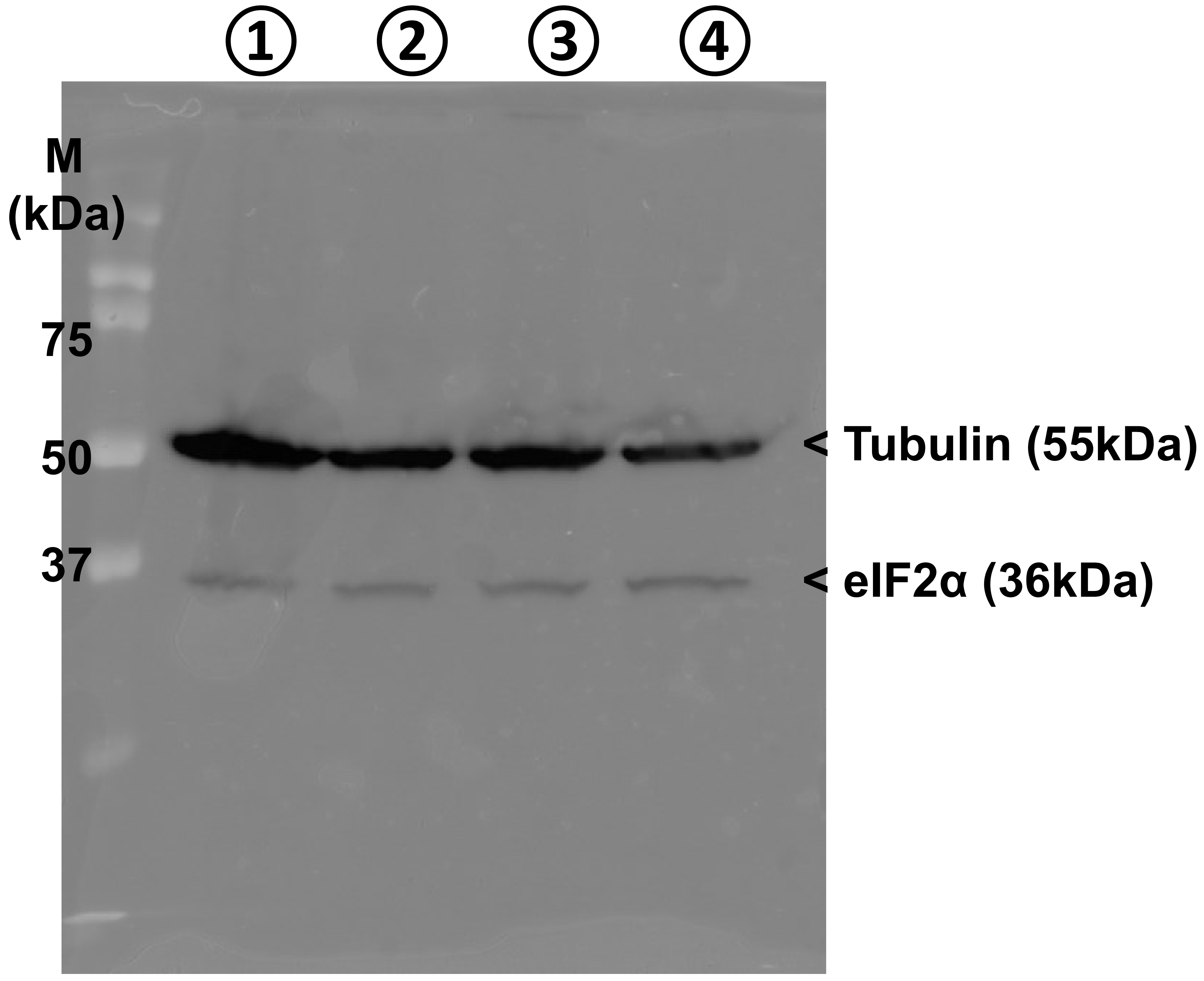

HAP1 ① WT, ② eIF2A-KO, ③ eIF2D-KO, ④ eIF2A-KO / eIF2D-KO cells

10^6 Cells loaded in each lane in 12.5% gel

First probed for anti-eIF2A, or -eIF2D, or -eIF2α antibodies.

And re-probed for anti-GAPDH or –tubulin antibodies.
